# Splenectomy as a diagnostic method in lymphoma-associated hemophagocytic lymphohistiocytosis of unknown origin

**DOI:** 10.1038/bcj.2016.125

**Published:** 2017-02-24

**Authors:** J Ma, Z Jiang, T Ding, H Xu, J Song, J Zhang, Y Xie, W Wang

**Affiliations:** 1 Department of Hematology, Huadong Hospital, Fudan University, Shanghai, China; 2 Department of General Surgery, Huadong Hospital, Fudan University, Shanghai, China; 3 Department of Hematology, Huashan Hospital, Fudan University, Shanghai, China; 4 Department of Infection, Huashan Hospital, Fudan University, Shanghai, China

Hemophagocytic syndrome, also known as hemophagocytic lymphohistiocytosis (HLH), is a rare and life-threatening disease caused by a highly active but ineffective immune response.^1^ Uncontrolled immune activation results in the typical clinical characteristics and laboratory findings of HLH, including high fever, pancytopenia, hepatosplenomegaly, liver dysfunction, coagulopathy, hyperferritinaemia and hemophagocytosis in the bone marrow or other organs. HLH may be divided into primary (familial) and secondary subtypes. Secondary HLH may develop following strong immunological stimuli such as a severe infection and malignancies especially lymphoma.^2, 3^ Indeed, in a review on 162 adult patients with HLH, 56.8% were cases of lymphoma-associated hemophagocytic syndrome (LAHS).^4^ The prognosis of both HLH and LAHS is poor.^5^ Without hematopoietic stem cell transplantation, the survival rate of patients with HLH is 0%.^6^ Furthermore, in a study on 69 patients with HLH (16 of whom had LAHS), the median survival time of patients with LAHS was only 37 days.^7^ Early diagnosis of the underlying condition, and especially of lymphoma, leads to better outcomes. However, the diagnosis of LAHS is often difficult because of the frequent lack of lymphadenopathy or solid tissue masses amenable to biopsy. Many patients with LAHS only have splenomegaly or high standardized uptake values (SUVs) of the spleen on FDG-PET/CT to suggest the diagnosis, and in such situations, splenectomy may be the only choice to secure a histological diagnosis. However, cytopenias and coagulopathy can make splenectomy a challenging and potentially hazardous undertaking. Here we report a retrospective analysis of 100 secondary HLH patients treated in two medical centers between 2012 and 2016. This study aimed to analyze the efficiency and safety of splenectomy in patients with secondary HLH.

We retrospectively reviewed the patients who were given the clinical code D76.1 (hemophagocytic lymphohistiocytosis) between January 2012 and April 2016 in two medical centers. The ethics committee from Huadong Hospital and Huashan Hospital approved this study. All patients provided informed consent. Pre-treatment evaluation consisted of a standard history and physical examination. Various pre-treatment tests were also performed, including routine blood tests (peripheral blood examination, biochemical tests and coagulation check), blood immunology, virology, bacteriology, bone marrow morphology, flow cytometry, bone marrow or lymph node tissue immunohistochemical staining, karyotype analysis, and CT or PET/CT scan. The diagnostic criteria of HLH was according to guidelines.^8, 9^ Sixty patients had neither palpable lymphadenopathy nor evidence of lymphoma in the bone marrow, but they all had a high FDG value of the whole spleen or some focus in the spleen. Among these patients, 25 underwent diagnostic splenectomy with blood product support in the form of plasma, platelet, and cryoprecipitate infusions, a further 35 patients rejected a splenectomy. Patients who did not undergo splenectomy were treated with etoposide and dexamethasone according to the international guidelines for the treatment of HLH 2004.^9^ Another 40 patients were diagnosed on the basis of lymph node biopsy or bone marrow biopsy and received standard chemotherapy.

For splenectomized patients, all tests repeated on days 1, 3 and 7 postoperatively. All patients must have received virology tests, such as an EBV-DNA quantitative test. Patients with a diagnosis of lymphoma received chemotherapy appropriate to their underlying pathology 1 week after the surgery. In contrast, HLH patients with Epstein–Barr virus (EBV) infection are typically treated with immunoglobulin post-operatively. The comparisons of the clinical features of the splenectomy and non-splenectomy groups, and the changes before and after splenectomy were performed using the *t*-test and *χ*^2^ test. Survival was assessed using the Kaplan–Meier curve method, and survival between the groups was compared using the log-rank test. The analysis of prognostic factors was performed using a linear regression model. A *P*<0.05 was considered statistically significant difference, SPSS software was explored to analyze the data.

The clinical features of patients in the splenectomy group and non-splenectomy group are comparable in Table 1. No significant differences of clinical features were evident (*P*>0.05). Among the HLH patients of indefinite underlying diseases, the white blood cell count was significantly higher in the non-splenectomy group compared with the splenectomy group (*P*=0.031), but no significant difference was observed between the two groups in any of the other factors (Table 1, *P*>0.05). Three days after splenectomy, many factors related to HLH got improvement, patients' body temperature was significantly lower (*P*=0.001). ALT and AST measurements were also significantly diminished (*P*=0.006 and *P*=0.01, respectively). The total white blood cell count was significantly higher following surgery (*P*=0.044), The TG level was lower (*P*=0.002). Although splenectomy has frequently been rejected in the treatment of HLH due to the perceived operative risk, we analyzed patients' death, which may be caused by operation in splenectomy group and non-splenectomy group, the survival data was not significantly different (*P*=0.510). This indicates that splenectomy itself does not shorten the survival time of patients with HLH.

We analyzed the survival data of patients with HLH of unknown origin with splenectomy. The follow-up data were obtained from 60 patients, 25 of them underwent splenectomy to obtain a pathological diagnosis. In the splenectomy group, median overall survival time was 130 (74.0–304.3) days, median progression free survival was 67 (11.67–203.9) days. In the non-splenectomy group, the median survival time was 89 (64.9–94.3) days, the median progression-free survival was 13.3 (5.0–25.2) days, patients with HLH of unknown origin in the splenectomy group had significantly improved overall survival compared with those in the non-splenectomy group and improved progression free survival (Figure 1a, *P*=0.001 vs 0.000). After splenectomy, a clear diagnosis could be determined: three patients were diagnosed with infection-associated HLH (two with underlying EBV infection, both of whom are still alive), six had underlying B-cell lymphoma (three of whom remain alive), seven had NKT cell lymphoma (two are still alive), and 10 had T-cell lymphoma (none survived, but one had a survival time exceeding three years). The 35 patients with HLH of unknown origin who did not undergo splenectomy all died. We performed a univariate analysis to evaluate the prognostic factors for HLH in the splenectomy group. The prognostic influence of several factors (including age, sex, white blood cell count, hemoglobin levels, platelet count, liver function, bilirubin levels, LDH, TG, Fbg, serum ferritin and underlying diagnoses) were analyzed. Only a lower platelet count (*r*=0.804, *P*=0.000) and a lower serum ferritin level (*r*=0.408, *P*=0.002) were predictive of poorer survival in the splenectomy group. But when we analyzed the effect of splenectomy in all HLH patients in our study, including the patients who make clear the diagnosis by lymphocyte or bone marrow biopsy, patients in the splenectomy group did not show improved overall survival and progression-free survival compared with those without splenectomy (Figure 1b, *P*=0.203 vs 0.143).

HLH is a serious condition with a high rate of mortality. Contemporary HLH treatment includes chemotherapy with etoposide (100–150 mg/m^2^ i.v.), and immunosuppressive drugs that specifically target the hyperactivated macrophages (including corticosteroids and intravenous immunoglobulin) and T lymphocytes (including corticosteroids and ciclosporin A).^9, 10^ However, immunochemotherapy is only temporarily effective in the control of HLH, determining the underlying cause of secondary HLH is necessary, but it is often difficult to secure the correct diagnosis as some patients do not have lymphomegaly, solid masses, or bone marrow involvement. Splenectomy was considered unsafe due to the poor clinical condition of HLH patients.^11, 12^ In this study, we examined 100 patients with HLH, if a definitive diagnosis could be made from lymphocyte and bone marrow diagnosis, then splenectomy was not necessary. We found that with appropriate transfusion support (plasma and platelet infusions), splenectomy was relatively safe in the HLH patients. As both clinical and laboratory parameters showed improvement post-operatively, which may be related to a reduced cytokine storm. Moreover, splenectomy created more chances for patients with HLH of unknown origin to find out the pathology. Splenectomy improved the survival of HLH patients with indefinite underlying diseases, but it did not prolong the survival of all patients with HLH, especially those with definite diagnosis. The optimal time for splenectomy under such circumstances was in the initial stages of HLH, which is in concordance with the prognostic factors in our study. In conclusion, our data support the hypothesis that splenectomy is a valid and safe diagnostic approach for secondary HLH of unknown origin, especially for those only having foci in the spleen. But splenectomy itself does not prolong survival in all patients with HLH, particularly ones with a definitive diagnosis. This report provides a rationale for the further mechanistic investigation of the role of splenectomy in this condition.

## Figures and Tables

**Figure 1:**
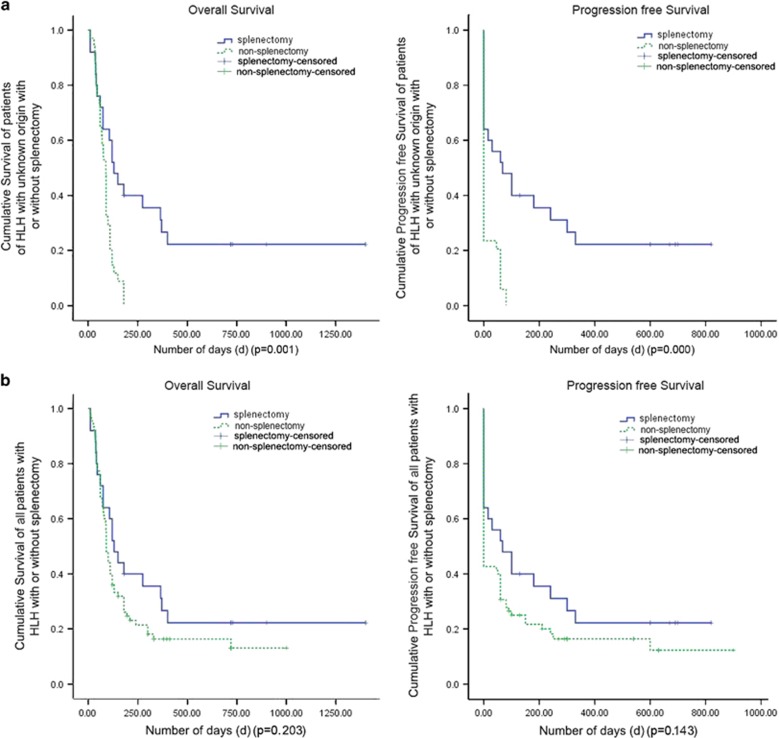
Kaplan–Meier overall survival (OS) and progression-free survival (PFS) curves of patients with HLH (**a**) Kaplan–Meier OS and PFS curves of patients of unknown origin with splenectomy (*n*=25) and without splenectomy (*n*=35) (*P*=0.001, left, overall survival vs *P*=0.000, right, progression-free survival). (**b**) Kaplan–Meier OS and PFS curves of all patients with HLH patients with splenectomy (*n*=25) and without splenectomy (*n*=75) (*P*=0.203, left, overall survival vs *P*=0.143, right, progression-free survival).

**Table 1:** Clinical features of patients with HLH in the splenectomy and non-splenectomy groups

	*All patients (n*=*100)*			*Patients of unknown origin (n*=*60)*		
	*Splenectomy*	*Non-splenectomy*	P-*value*	*Splenectomy*	*Non-splenectomy*	P-*value*
Sex, n (F/M)	17/8	40/35	0.20	17/8	14/21	0.051
Age, years (range)	45.8 (19–66)	46.4 (2–67)	0.051	45.8 (19–66)	49.0 (34–67)	0.05
Temperature, °C (range)	39.7 (38.0–40.4)	39.0 (38.3–40)	0.67	39.7 (38.0–40.4)	39.1 (38.5–40)	0.60
WBC, × 10^9^ cells/l (range)	2.75 (0.6–9.1)	3.66 (0.5–10.16)	0.071	2.75 (0.6–9.1)	4.68 (0.7–6.4)	0.031
PLT, 10^9^ cells/l (range)	61.2 (4–305)	51.4 (5–197)	0.143	61.2 (4–305)	44.1 (30.8–59)	0.057
TB, μmol/l (range)	52.7 (7.4–230.7)	49.0 (18.9–332.7)	0.472	52.7 (7.4–230.7)	43.7 (27–332)	0.729
DB, μmol/l (range)	37.3 (3.4–218)	39.2 (11.8–272.3)	0.286	37.3 (3.4–218)	36.8 (13.4–272)	0.414
TG, mmol/l (range)	3.23 (2.0–6.7)	3.21 (3.2–6.6)	0.284	3.23 (2.0–6.7)	3.0 (2.8–5.1)	0.425
Fbg, g/l (range)	1.78 (0.58–3)	1.32 (0.41–1.8)	0.773	1.78 (0.58–3)	1.2 (0.6–1.8)	0.228
Ferritin, ng/l (range)	3571 (2498–4956)	4448 (3151–6669)	0.556	3571 (2498–4956)	3676 (3151–5788)	0.640
LDH, U/l (range)	931 (68–1205)	2799 (1037–6044)	0.256	931 (686–1205)	1239 (1037–3499)	0.118
Underlying diagnosis, n:			0.10			
EBV infection	2	9		2	Unkown	
Autoimmune disease	0	2		0	Unkown	
B-cell lymphoma	6	10		6	Unkown	
NKT cell lymphoma	7	7		7	Unkown	
T-cell lymphoma	10	12		10	Unkown	
Unknown origin	0	35			35	
PS (range)	1 (0–2)	1 (1–2)	0.57	1 (0–2)	1 (1–2)	0.30

Abbreviations: DB, direct bilirubin; EBV, Epstein–Barr virus; Fbg, fibrinogen; LDH, lactate dehydrogenase; PLT, platelet; PS, physical stage TB, total bilirubin; TG, triglycerides; WBC, white blood count.
